# Study on the mechanical properties of rock with cross fracture under biaxial compression cyclic loading and unloading

**DOI:** 10.1371/journal.pone.0352591

**Published:** 2026-08-14

**Authors:** Liangxiao Xiong, Haijun Chen, Hanqiang Wang, Zhongyuan Xu, Xin Zhang, Yi Tao

**Affiliations:** 1 School of Civil Engineering and Architecture, East China Jiaotong University, Nanchang, China; 2 State Key Laboratory of Geohazard Prevention and Geoenvironment Protection, Chengdu University of Technology, Chengdu University of Technology, Chengdu, Sichuan, China; 3 Geotechnical Engineering Department, Nanjing Hydraulic Research Institute, Nanjing, China; 4 The First Geological Brigade of Shandong Provincial Bureau of Geology & Mineral Resources (Shandong No.1 Geological & Mineral Exploration Institute), Jinan, China; 5 Faculty of Geosciences and Environmental Engineering, Southwest Jiaotong University, Chengdu, China; 6 Qingdao Zhonghe Eco-Technology Research Institute Co. Ltd., Qingdao, China; China Construction Fourth Engineering Division Corp. Ltd, CHINA

## Abstract

In this study, biaxial compression cyclic loading and unloading tests are performed on rock-like samples that contain a single group of cross cracks. The effects of angle *α* between the main crack and load direction, the angle between the main and secondary cracks, and the number of cycles of biaxial compression cyclic loading and unloading on the coabsmpressive strength and crack evolution of the samples are investigated. The effects of the number of groups of cross cracks and number of cycles of cyclic loading and unloading on the compressive strength and crack evolution are then examined. When both angle *β* between the primary and secondary prefabricated cracks and the lateral pressure are similar, the compressive strength of the specimen first decreases and then increases with *α* from 0° to 90°. When *α* is the same, the lateral pressure is the same, and *β* between the primary and secondary prefabricated cracks increases from 0° to 90°, the compressive strength of the sample does not significantly change. When *α* is the same, *β* is the same, and the lateral pressure is the same, the compressive strength of the specimen after cyclic loading and unloading gradually decreases with increasing number of cyclic loading and unloading. When both *α* and the lateral pressure are the same, the compressive strength of the specimen gradually decreases with increasing number of cross cracks in the specimen. Main crack is the main controlling factor of the strength and crack propagation of specimens with a single group of cross cracks. The interaction among multiple groups of intersecting cracks changes the state of the local stress field, and the crack propagation is more complex than that in a single group of cross cracks.

## 1 Introduction

Rock masses in natural geological environments are typically subjected to triaxial stress, whereas underground engineering structures mostly experience biaxial stress conditions, such as the surface rock after tunnel and slope excavation, which is under biaxial compression. Therefore, conducting a biaxial compression test on rock specimens with cracks has practical engineering significance.

Zhao et al. [1] analyzed and compared the cracking behavior of rock-like specimens with single flaws under uniaxial and biaxial compression and reported that the confining pressure changed their boundary condition and cracking characteristics under biaxial compression. Han et al. [2] investigated the effect of joint dip angle on the mechanical behavior of infilled jointed rock mass under uniaxial and biaxial compression, and the biaxial compressive strength exhibited a V-shaped change with the increase in the dip angle. Bi et al. [3] investigated the failure process of rock-like materials subjected to biaxial compressive loads using numerical code GPD3D and reported that the secondary cracks continued growing in the samples with increase in lateral stress. Wang et al. [4] prepared rock-like materials with two preexisting fissures, performed biaxial compression tests on these specimens and reported that the rock-bridge coalescence mode could be generally classified into three categories. Wang et al. [5,6] conducted biaxial compression experiments on 60 specimens and reported that three main rock-failure patterns appeared in rock bridges. Du et al. [7] conducted a series of biaxial compression and biaxial fatigue tests to investigate the mechanical behavior of marble and sandstone under biaxial confinements. The experimental results demonstrated that the biaxial compressive strength of rocks under biaxial compression first increased and subsequently decreased with the increase in the intermediate principal stress. Zhang et al. [8] discovered that compared with the uniaxial condition, a rock sample with a closed straight crack could withstand additional stress under in-plane biaxial compression. Zhao et al. [9] conducted a series of biaxial compression tests with a lateral stress of 2.0 MPa on rock-like specimens that contained two flaws. Seven basic crack types were identified, and 10 patterns of crack coalescence were observed from the biaxial compression experiments on flawed specimens. Huang et al. [10] performed spallation experiments on mudstone under biaxial compression. The results demonstrated that the stress–strain curve of mudstone under biaxial compression exhibited significant strain rebound. Zhang et al. [11] investigated the influence of lateral pressure on the mechanical behavior of four typical rock types under biaxial compression. The results indicated that the brittleness and burst proneness of rock or coal material was stronger than those of gypsum materials because of differences in mineral compositions and structures.

The abovementioned researchers primarily performed biaxial compression tests on intact rock specimens or specimens with parallel cracks. In actual rock engineering, cross cracks often occur. Liu et al. [12] performed an experimental study on molded gypsum specimens with different crack geometries (T- and X-shaped cross cracks) under biaxial compression, and nine crack types were detected. Liu et al. [13] reported that the width and length of new fractures under biaxial compression were smaller than those under uniaxial-compression conditions. However, Liu et al. [12,13] primarily conducted biaxial compression tests on samples with a single group of cross cracks, whereas actual rock-mass engineering often contains multiple groups of cross fractures.

Engineering rock masses are subjected not only to bear static loads but also to varying degrees of cyclic loading, such as tectonic geological forces and weathering-induced erosion under natural conditions. Tunnel excavation can impose cyclic loads on surrounding rock masses, while the foundations of critical infrastructure like highways, railways, and bridges are subjected to periodic or cyclic loads from vehicles and blasting. These cyclic loads promote the progressive expansion and interconnection of fractures within the rock mass, ultimately resulting in failure. Therefore, studying the mechanical properties and failure mechanisms of fractured rock masses under cyclic loading is crucial for understanding the failure mechanisms and long-term stability of engineering rock masses. Currently, research on the mechanical characteristics of fractured rock masses under cyclic loading remains relatively scarce.

In the present study, biaxial compression cyclic loading and unloading tests are conducted on rock-like samples that contain a single group of cross cracks. The effects of the angle between the main cracks and load direction, that between the main and secondary cracks, and the number of cycles of biaxial compression cyclic loading and unloading on the compressive strength and crack evolution of the samples are investigated. Moreover, the effects of the number of cross-crack groups and number of cycles of cyclic loading and unloading on the compressive strength and crack evolution are studied.

## 2 Test scheme

### 2.1 Sample preparation

**Fig 1:**
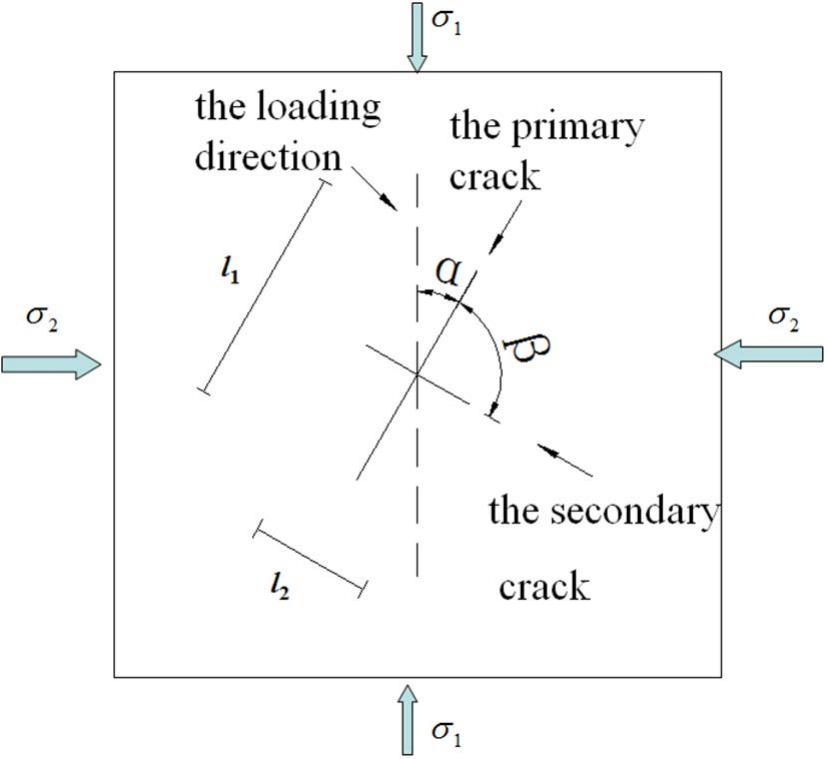
Schematic of the stress in the specimen with cross crack under biaxial compression.

Biaxial compression cyclic loading and unloading tests were performed on artificial rock-like samples with cracks. This type of rock samples was composed of cement mortar. Due to the difficulty in forming fractures on natural rock specimens, cement mortar is commonly used as a rock-like material, with fractures created in the cement mortar specimens. Xiong et al. [14–16], employed cement mortar specimens with fractures to simulate fractured rock, conducting uniaxial compression tests and shear tests. Therefore, cement mortar specimens can be used to simulate the mechanical properties of rock. However, when using cement mortar specimens to simulate rock’s mechanical properties, there remains a certain discrepancy between the mechanical characteristics of the cement mortar and those of rock, typically addressed by adhering to the principles of similarity in specimen fabrication. Given that the biaxial compression testing machine employed in this study has a maximum axial load capacity of 600 kN, cement mortar specimens with low uniaxial compressive strength were selected for the experiments. Therefore, the cement was 325 Portland cement, the sand was medium standard sand, and the water–cement ratio was 0.65. The sample was a cube sample that was 100-m long, 100-mm high, and 100-mm wide. First, the cement mortar was poured in the cube test mold and then vigorously vibrated. After 12 h, a plastic sheet was inserted based on the crack position shown in Fig 1 and then pulled out from the plastic sheet after 12 h to form an empty crack in the sample. Fig 1 shows the stress diagram of the specimen with cross cracks under biaxial compression.

The specimens were cured in the curing room at 20 °C and 95% relative humidity for 28 days before they were tested.

**Fig 2:**
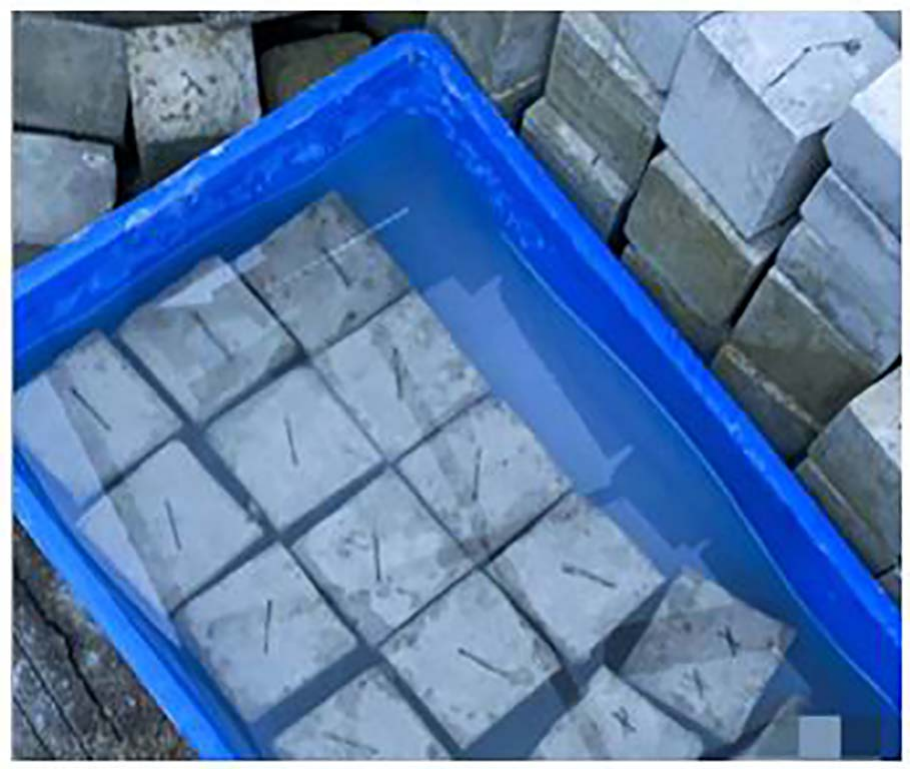
Test specimen after molding.

Fig 1 and the later part show that the angle between the main crack and direction of maximum principal stress *σ*_1_ is denoted as *α*. The angle between the main fracture and secondary fracture is denoted as *β*. The length of the main fracture is denoted as *l*_1_, whereas that of the secondary fracture is denoted as *l*_2_. *l*_1_ and *l*_2_ remained unchanged at 40 and 20 mm, respectively. The crack width is 1 mm。The formed specimen is shown in Fig 2.

### 2.2 Fracture combination form

In this work, the test was divided into four groups based on the combination of cracks. The first group comprised one group of intersecting fissures. Fig 1 shows the diagram with the fissures (see Table 1 for details).

**Table 1:** Test grouping with a single group of cross cracks.

Study	the length *l*_1_/ mm	the length *l*_2_/ mm	the angle *α/* °	the angle *β/* °	Number of specimens
1	40	20	0	0, 30, 45, 60 and 90	15
2	40	20	30	0, 30, 45, 60 and 90	15
3	40	20	45	0, 30, 45, 60 and 90	15
4	40	20	60	0, 30, 45, 60 and 90	15
5	40	20	90	0, 30, 45, 60 and 90	15

**Fig 3:**
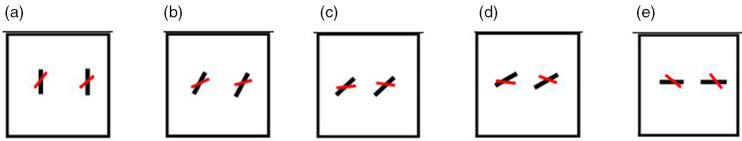
Diagram of the specimens with two groups of cross cracks when *β* = 30°. (a) *α* = 0° (b) *α* = 30° (c) *α* = 45° (d) *α* = 60° (e) *α* = 90°.

**Fig 4:**
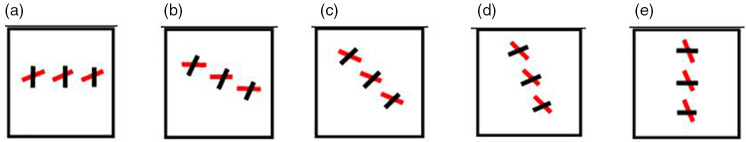
Diagram of the specimens with three groups of cross cracks when *β* = 60°. (a) *α* = 0° (b) *α* = 30° (c) *α* = 45° (d) *α* = 60° (e) *α* = 90°.

**Fig 5:**
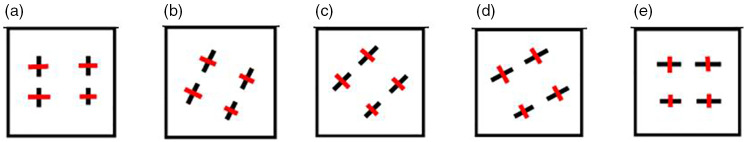
Diagram of the specimens with four groups of cross cracks when *β* = 90°. (a) *α* = 0° (b) *α* = 30° (c) *α* = 45° (d) *α* = 60° (e) *α* = 90°.

Diagram of the specimens with two groups of cross cracks when *β* = 30° is shown in Fig 3, diagram of the specimens with three groups of cross cracks when *β* = 60° is shown in Fig 4, and diagram of the specimens with four groups of cross cracks when *β* = 90° is shown in Fig 5. Due to the variety of experimental configurations, in order to reduce the number of test conditions, *β* containing two sets of intersecting cracks, three sets of intersecting cracks, and four sets of intersecting cracks will be temporarily randomly selected.

### 2.3 Test instrument

A biaxial-compression test was performed on a biaxial compression testing machine. The maximum axial load that can be applied to the testing machine is 600 kN, and the maximum lateral load that can be applied is 300 kN.

### 2.4 Loading method

The tests performed in this study included biaxial compression and biaxial compression cyclic loading and unloading tests.

The biaxial-compression test steps are described as follows. (1) First, axial and side pressure were simultaneously applied at the set values and kept for 1 min. (2) The lateral pressure was kept unchanged, and axial stress was continually applied until the specimen was destroyed.

In the biaxial cyclic loading and unloading tests, the uniaxial compression test was first performed to obtain the uniaxial compressive strength, which provided a basis for determining the loading and unloading positions in the biaxial compression cyclic loading and unloading tests. In this study, three standard cylindrical specimens were used for the uniaxial compression test, and the average uniaxial compressive strength of the specimens was 24.14 MPa. The lateral pressure was constant, and only axial cyclic loading and unloading were performed on the specimen during the biaxial cyclic loading and unloading tests.

**Fig 6:**
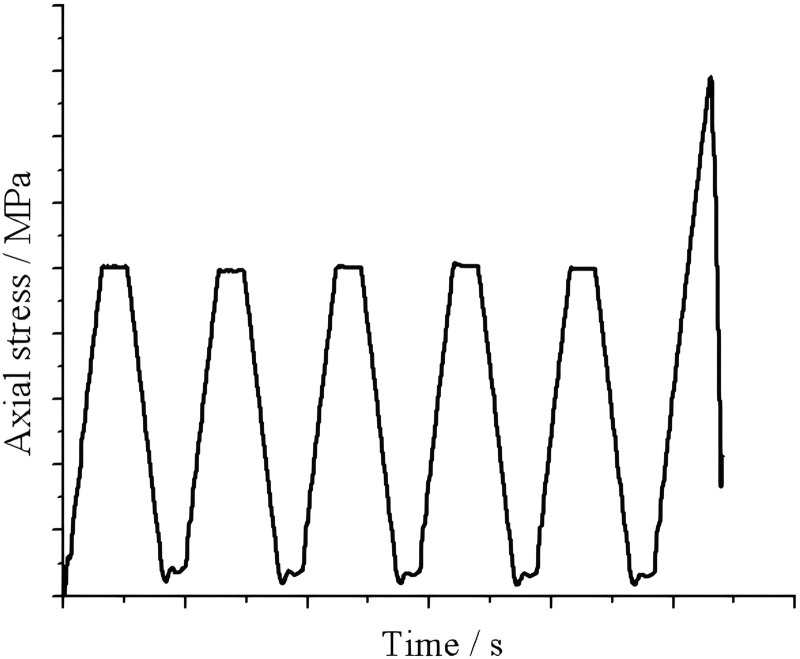
Relationship curve between the axial pressure and time during loading and unloading of the biaxial-compression cycle.

The specific steps of the biaxial- cyclic loading and unloading tests are described as follows. (1) Axial and lateral pressure were applied at the same loading rate. When the lateral pressure reached the predetermined setting value, it remained unchanged. Axial pressure was continually applied up to 12 MPa and kept unchanged for 1 min. (2) The axial pressure started to be reduced to a predetermined value of lateral pressure, kept unchanged for 1 min, and continually applied up to 12 MPa. It was then maintained for 1 min and subsequently continually unloaded to a predetermined value of lateral pressure. The cycle was repeated five times. (3) After the end of the five cycles, the axial pressure was continuously directly loaded until damage occurred. The test was then stopped. Fig 6 shows the relationship between the stress and time under axial loading. During cycle loading and unloading biaxial compression, the lateral pressure is maintained at 1MPa or 2MPa.

In the biaxial -compression and biaxial compression cyclic loading and unloading tests, two types of lateral pressure were included (i.e., 1 and 2 MPa). The loading rate of the axial and lateral pressure was 0.5 MPa/s.

## 3 Analysis of the biaxial compression cyclic loading and unloading test results

### 3.1 Analysis of the compressive strength of the biaxial compression test

Table 2 lists the test values of the compressive strength of the specimen with a single group of cross cracks under biaxial compression.

**Table 2:** Test values of the compressive strength of the specimen containing a single group of cross cracks under biaxial compression.

Inclined angle *α* of main crack */* °	Lateral pressure is 1 MPa					Lateral pressure is 2 MPa				
	The angle between the main fracture and the secondary fracture *β/* °					The angle between the main fracture and the secondary fracture *β/* °				
	0°	30°	45°	60°	90°	0°	30°	45°	60°	90°
0°	20.00	19.58	18.47	17.08	18.40	20.81	20.60	19.07	17.54	18.36
30°	16.52	16.07	16.82	17.02	15.77	17.75	17.01	17.44	17.24	16.12
45°	14.74	14.62	14.83	14.73	15.25	15.61	14.69	15.10	15.30	15.50
60°	14.28	12.83	13.00	12.73	12.96	14.59	13.06	12.95	13.36	13.57
90°	15.23	14.60	14.70	15.05	15.38	16.22	14.99	16.01	16.12	16.63

When inclined angle *α* is 0°, the compressive strength of the specimen first decreases and then increases with the increase in angle *β* from 0° to 90°. When *α* is 30°, 45°, 60°, and 90°, the compressive strength of the specimen slightly changes with the increase in *β* from 0° to 90°. The change in *β* has little effect on the strength of the specimen.

When *β* remains unchanged, the compressive strength of the specimen first decreases and then increases with the increase in *α* from 0° to 90°. The compressive strength of the specimen reaches the minimum value when *α* = 60°. This is mainly due to the maximum shear stress is concentreated along the main crack at this time, which is consistent with many existing experimental results. The influence of the change in *α* on the compressive strength of the specimen is obvious. The reason is that the length of the main fracture is 40 mm and that of the secondary fracture is 20 mm. When the inclined angle of the main fracture remains unchanged, changing the angle between the main and secondary cracks induces a small effect on the compressive strength of the sample.

Comparing fractured rock specimens with a crack angle of β = 0° (single-crack specimens) with other angles show that the appearance of secondary cracks affects the strength variation law of the fractured rock mass. In addition, some samples with intersecting cracks have significantly higher strength than single crack samples. The deformation and fracture of secondary cracks to some extent reduce the stress concentration at the end of the main crack and improve the strength of the entire model.

**Fig 7:**
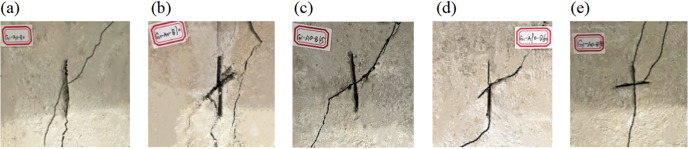
Failure pattern of the specimen with a single group of cross cracks under biaxial compression at lateral pressure of 1 MPa and *α* = 0°. (a) *α* = 0°, *β* = 0° (b) *α* = 0°, *β* = 30° (c) *α* = 0°, *β* = 45° (d) *α* = 0°, *β* = 60° (e) *α* = 0°, *β* = 90°.

**Fig 8:**
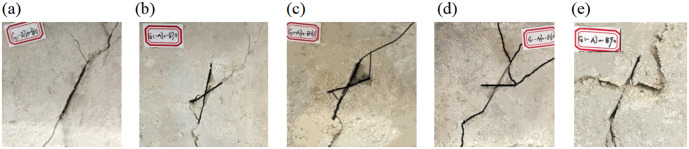
Failure pattern of the specimen with a single group of cross cracks under biaxial compression when the lateral pressure is 1 MPa and *α* = 30°. (a) *α* = 30°, *β* = 0° (b) *α* = 30°, *β* = 30° (c) *α* = 30°, *β* = 45° (d) *α* = 30°, *β* = 60° (e) *α* =30°, *β* = 90°.

**Fig 9:**
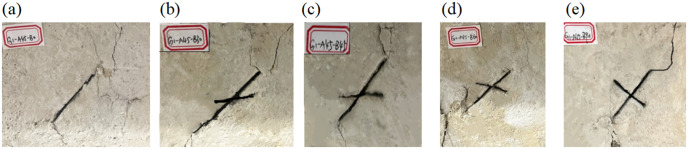
Failure pattern of the specimen with a single group of cross cracks under biaxial compression when the lateral pressure is 1 MPa and *α* = 45°. (a) *α* = 45°, *β* = 0° (b) *α* = 45°, *β* = 30° (c) *α* = 45°, *β* = 45° (d) *α* = 45°, *β* = 60° (e) *α* = 45°, *β* = 90°.

**Fig 10:**
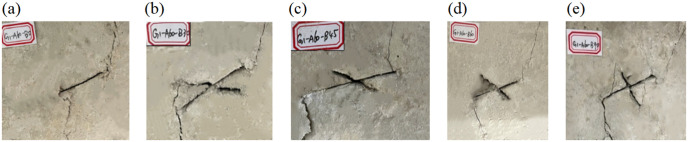
Failure pattern of the specimen with a single group of cross cracks under biaxial compression when the lateral pressure is 1 MPa and *α* = 60°. (a) *α* = 60°, *β* = 0° (b) *α* = 60°, *β* = 30° (c) *α* = 60°, *β* = 45° (d) *α* = 60°, *β* = 60° (e) *α* = 60°, *β* = 90°.

**Fig 11:**
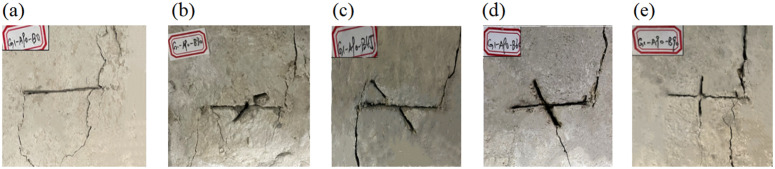
Failure pattern of the specimen with a single group of cross cracks under biaxial compression when the lateral pressure is 1 MPa and *α* = 90°. (a) *α* = 90°, *β* = 0° (b) *α* = 90°, *β* = 30° (c) *α* = 90°, *β* = 45° (d) *α* = 90°, *β* = 60° (e) *α* = 90°, *β* = 90°.

The failure pattern of the specimen that contains a single group of cross cracks under biaxial compression when the lateral pressure is 1 MPa is shown in Figs 7–11.

When *α* = 0° and *β* = 0°, the primary prefabricated crack exhibits no obvious effect on the fracture of the rock sample under biaxial compression. The stress–concentration area is primarily located at the tip of the secondary prefabricated crack. The model demonstrates an overall shear compression failure.

When *β* between the primary and secondary prefabricated cracks increases, the tip of the secondary prefabricated crack exhibits obvious stress concentration and starts to crack and generates wing cracks, which continue to expand with the increase in the load.

When *α* = 0° and *β* = 90°, wing cracks appear at the tips of the primary and secondary prefabricated cracks.

When *α* = 30° and *β* = 0°, a secondary coplanar crack is generated at the tip of the main prefabricated crack and propagates along the direction of the main prefabricated crack.

When *α* = 30° and *β* > 0°, although the generation of wing cracks is controlled by the primary cracks, the wing cracks at the end of the primary cracks overlap and penetrate into the wing cracks at the end of the secondary cracks because of the effect of secondary cracks.

When *α* = 45° and the range of *β* is from 0° to 90°, secondary wing cracks are generated at the tip of the primary crack, and the wing cracks are controlled by the primary crack.

When *α* = 60° and the range of *β* is from 0° to 60°, a secondary wing crack is generated at the tip of the main prefabricated crack, and the wing crack extends to the upper and lower surfaces of the specimen.

When *α* = 60° and *β* = 90°, secondary wing cracks are generated at the tip of the secondary prefabricated crack, and the wing cracks at the end of the primary prefabricated crack overlap and penetrate into the wing cracks at the end of the secondary prefabricated crack.

When *α* = 90° and the range of *β* is from 0° to 90°, a secondary wing crack is generated at the tip of the main prefabricated crack, and the wing crack extends and penetrates along the vertical loading direction.

Table 3 lists the test values of the compressive strength of the specimens with multiple groups of cross cracks under biaxial compression.

**Table 3:** Test value of compressive strength of specimen with multiple groups of cross cracks under biaxial compression (MPa).

Inclined angle of main fracture*α* */* °	Lateral pressure is 1 MPa			Lateral pressure is 2 MPa		
	Number of cross cracks			Number of cross cracks		
	Two groups of cross cracks	Three groups of cross cracks	Four groups of cross cracks	Two groups of cross cracks	Three groups of cross cracks	Four groups of cross cracks
0°	18.81	16.44	15.82	19.55	17.06	16.43
30°	15.64	15.83	14.93	16.22	16.43	15.50
45°	13.65	13.72	12.74	14.14	14.25	13.21
60°	12.68	12.01	11.61	13.10	12.48	12.06
90°	13.40	14.77	15.13	13.94	15.29	15.70

When *α* varies, the compressive strength of the specimen gradually decreases with increase in the number of groups of cross cracks.

When the number of crack groups is the same, the compressive strength of the specimen first decreases and then increases with the increase in *α* from 0° to 90°. The compressive strength of the specimen reaches a minimum value when *α* = 60°. This is mainly due to the maximum shear stress is concentrated along the main crack at this time, which is consistent with many experimental results currently available. The influence of the change in *α* on the compressive strength of the specimen is obvious. The compressive strength of the specimen increases with the increase in the lateral pressure when the specimen contains the same crack combination.

### 3.2 Analysis of the compressive strength of biaxial-compression cyclic loading and unloading

Table 4 lists the test values of the compressive strength of the specimen that contains a single group of cross cracks after five times of biaxial compression cyclic loading and unloading.

**Table 4:** Test values of the compressive strength of the specimen that contains a single group of cross cracks after five times of biaxial-compression cyclic loading and unloading (MPa).

Inclined angle *α* the main crack of/ °	Lateral stress is 1 MPa					Lateral stress is 2 MPa				
	The angle *β* between the main and the secondary cracks*/* °					The angle *β* between the main and the secondary cracks*/* °				
	0°	30°	45°	60°	90°	0°	30°	45°	60°	90°
0°	19.68	18.88	17.28	16.72	18.01	20.64	19.36	17.76	17.5	18.72
30°	16.02	15.63	14.52	16.64	14.76	16.68	16.08	15.6	17.36	15.12
45°	14.64	14.04	14.42	14.28	14.76	15.96	14.64	15.48	14.40	15.28
60°	14.28	12.18	14.04	12.16	12.93	15.12	13.28	15.03	13.02	13.92
90°	15.12	14.28	14.64	14.96	15.24	15.16	14.80	14.92	15.44	16.16

Under biaxial compression cyclic loading and unloading conditions, the change trend of the compressive strength of the specimen with a single group of cross cracks varies with *α* and *β*. The lateral pressure is the same as that of the previous biaxial compression. When both *α*, *β*, and the lateral pressure are the same, the compressive strength of the specimen after five cycles of loading and unloading is less than that under biaxial compression.

**Fig 12:**
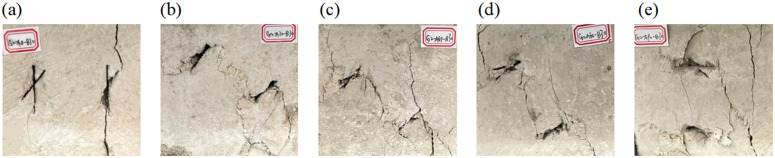
Failure pattern of the specimen with two groups of cross cracks under biaxial compression when the lateral pressure is 1 MPa. (a) *α* = 0° (b) *α* = 30° (c) *α* = 45° (d) *α* = 60° (e) *α* = 90°.

**Fig 13:**
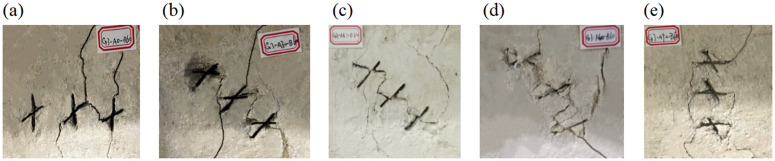
Failure pattern of the specimen with three groups of cross cracks under biaxial compression when the lateral pressure is 1 MPa. (a) *α* = 0° (b) *α* = 30° (c) *α* = 45° (d) *α* = 60° (e) *α* = 90°.

**Fig 14:**
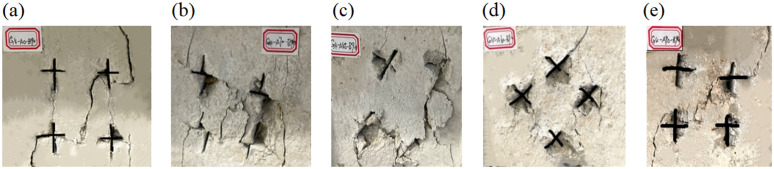
Failure pattern of the specimen with four groups of cross cracks under biaxial compression when the lateral pressure is 1 MPa. (a) *α* = 0° (b) *α* = 30° (c) *α* = 45° (d) *α* = 60° (e) *α* = 90°.

Figs 12–14 show the failure pattern of the specimen with multiple groups of cross cracks under biaxial compression when the lateral pressure is 1 MPa.

Compared with the single group of cross cracks, the interaction among multiple groups of cross cracks changes the state of the local stress field, and the crack propagation is more complex. Because of the different arrangement and distribution of multiple groups of cross cracks, the interaction among the cracks is different. The local stress field changes the crack-propagation mode. Furthermore, certain cracks in the rock-bridge area are connected, which makes the crack-propagation form and stress characteristics more complicated. The larger the spacing between cracks, the weaker the interaction between cracks.

Compared with single-group cross crack specimens, multi-group cross crack specimens exhibit an earlier onset time, earlier appearance of large-scale cracks, and lower peak intensity than single group cross crack specimens. These results indicate that the crack density has a significant weakening effect on the mechanical properties of the specimen.

Table 5 lists the test values of the compressive strength of the specimens with multiple groups of cross cracks after five times of biaxial-compression cyclic loading and unloading.

**Table 5:** Test value of the compressive strength of specimens with multiple groups of cross cracks after five times of biaxial-compression cyclic loading and unloading (MPa).

Inclined angle *α* of the main crack*/* °	Lateral stress is 1 MPa			Lateral stress is2 MPa		
	Number of cross cracks			Number of cross cracks		
	Two groups of cross cracks	Three groups of cross cracks	Four groups of cross cracks	Two groups of cross cracks	Three groups of cross cracks	Four groups of cross cracks
0°	18.04	16.44	15.44	19.68	16.81	16.42
30°	15.12	14.56	15.01	16.32	15.16	15.83
45°	13.62	12.52	12.24	14.76	13.24	13.92
60°	11.91	11.74	11.02	13.28	12.48	12.52
90°	13.16	14.40	15.32	14.16	15.32	15.76

Under biaxial compression cyclic loading and unloading conditions, the variation pattern of the compressive strength of the specimens with multiple groups of cross cracks varies with *α*. The number of groups of cross fractures and the lateral pressure are the same as that under biaxial compression. When *α*, the number of cross cracks, and the lateral pressure are the same, the compressive strength of the specimen after five cycles of loading and unloading is less than that under biaxial compression.

## 4 Determination of numerical simulation parameters

**Fig 15:**
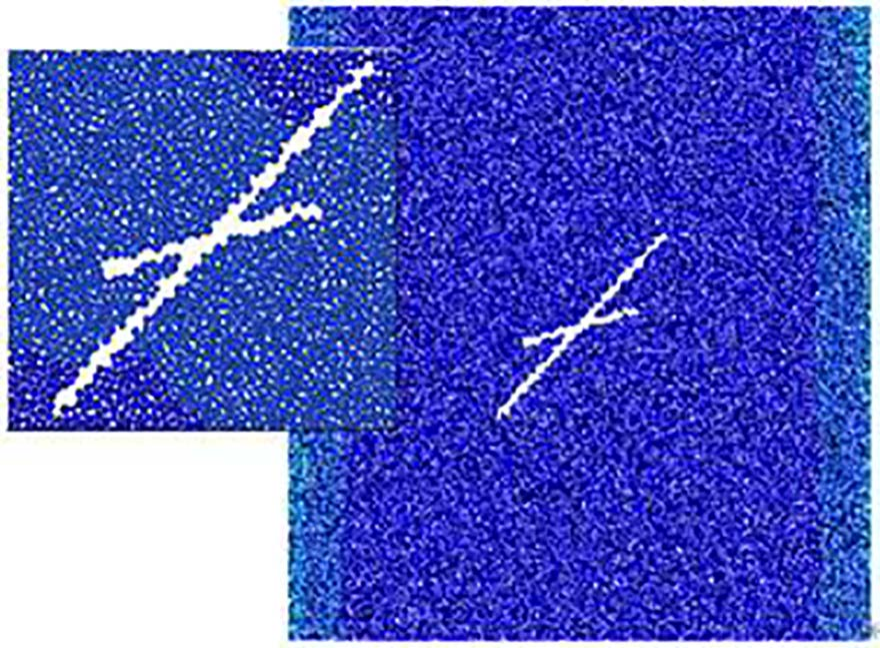
Diagram of a numerical model of the cross fracture.

The fish language provided by the PFC2D program is used to establish the numerical-simulation calculation model of the fractured rock. A 100 mm × 100 mm numerical model is established based on the relevant physical parameters of the intact test block. The particles are uniformly distributed, and the particle size is 0.3–0.5 mm. A total of 17991 spheres are generated from the intact sample, and the generated particles are constrained by four boundary walls. After the intact model is generated, the side walls and particles outside the specified size are removed. An external displacement is applied to the wall at the top of the model along the axial direction until the rock mass is damaged. The displacement rate is 0.05 mm/s. Fig 15 shows the diagram of the model, whereas Table 6 lists the mesoscopic parameters of the intact specimen.

**Table 6:** Meso parameters of a complete specimen.

density (kg·m^-3^)	minimum particle size (mm)	maximum particle size (mm)	friction coefficient
1960	0.3	0.5	0.5
Parallel bond modulus (GPa)	stiffness ratio of parallel bonding	mean strength of normal bond (MPa)	mean strength of tangential bond
1.20	1	21.0 ± 2	21.0 ± 2

Although the use of PFC2D can simulate the crack evolution behavior of the specimen, the limitation of PFC2D model is that it cannot obtain the stress field of the entire model.

## 5 Analysis of the numerical-simulation results of the biaxial-compression cyclic loading and unloading

### 5.1 Analysis of the compressive strength of the biaxial-compression test results

**Fig 16:**
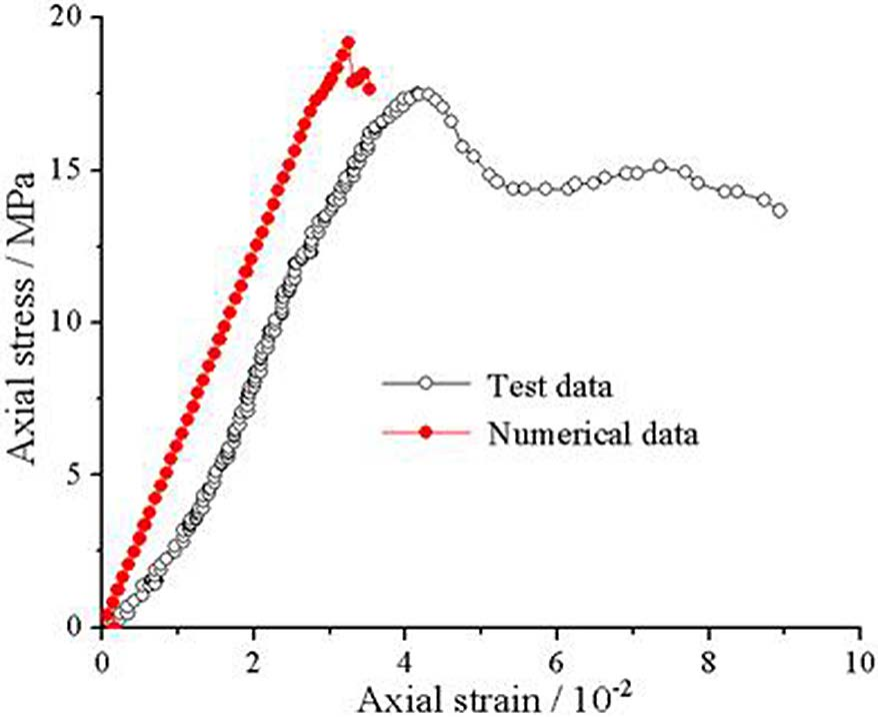
Comparison results of the axial stress–strain curves obtained from the experiment and numerical simulation under biaxial compression.

The mechanical properties of the specimen with a single group of cross cracks under biaxial compression are numerically simulated. Angle *α* of the specimen is 0°, angle *β* is 45°, and the lateral pressure is 1 MPa. Fig 16 shows the comparison results of the axial stress–strain curves obtained from the test and numerical simulation.

The stress–strain curves of the indoor test results are in good agreement with the numerical simulation test results. Hence, the PFC numerical calculation model and calculation parameters presented in this paper can be used to calculate the mechanical properties under biaxial compression.

Table 7 lists the numerical-simulation values of the compressive strength of the sample that contains a single group of cross cracks under biaxial compression, and Table 8 lists those of the compressive strength of the specimen that contains multiple groups of cross cracks under biaxial compression.

**Table 7:** Numerical-simulation values of the compressive strength of specimen with a single group of cross cracks under biaxial compression (MPa).

Inclined angle *α* of the main crack*/* °	Lateral stress is 1 MPa				
	The angle *β* between the main and the secondary cracks*/* °				
	0°	30°	45°	60°	90°
0°	20.52	20.34	20.01	19.06	20.13
30°	17.84	16.87	17.66	17.87	16.55
45°	15.97	15.95	16.17	16.06	16.01
60°	15.09	14.07	14.25	13.96	13.60
90°	16.49	15.33	15.43	15.80	16.14

**Table 8:** Numerical simulation value of the compressive strength of specimens with multiple groups of cross cracks under biaxial compression (MPa).

Inclined angle *α* of the main crack*/* °	Lateral stress is 1 MPa		
	Number of cross cracks		
	Two groups of cross cracks	Three groups of cross cracks	Four groups of cross cracks
0°	19.74	17.06	16.43
30°	16.38	16.12	15.20
45°	14.28	13.97	12.95
60°	13.23	12.48	12.06
90°	14.07	14.85	15.25

According to the numerical simulation results, when *β* remains unchanged, the compressive strength of the specimen first decreases and then increases with the increase in *α* from 0° to 90°. The compressive strength of the specimen reaches the minimum when *α* is 60°, and the influence of the change in *α* on the compressive strength of the specimen is obvious. The rule is the same as that obtained from the experiment.

When *α* of the main crack remains unchanged, the compressive strength of the specimen gradually decreases with the increase in the number of cross-crack groups according to the numerical-simulation results. The rule is the same as that obtained from the experiment.

### 5.2 Compressive strength analysis of the biaxial compression cyclic loading and unloading test results

**Fig 17:**
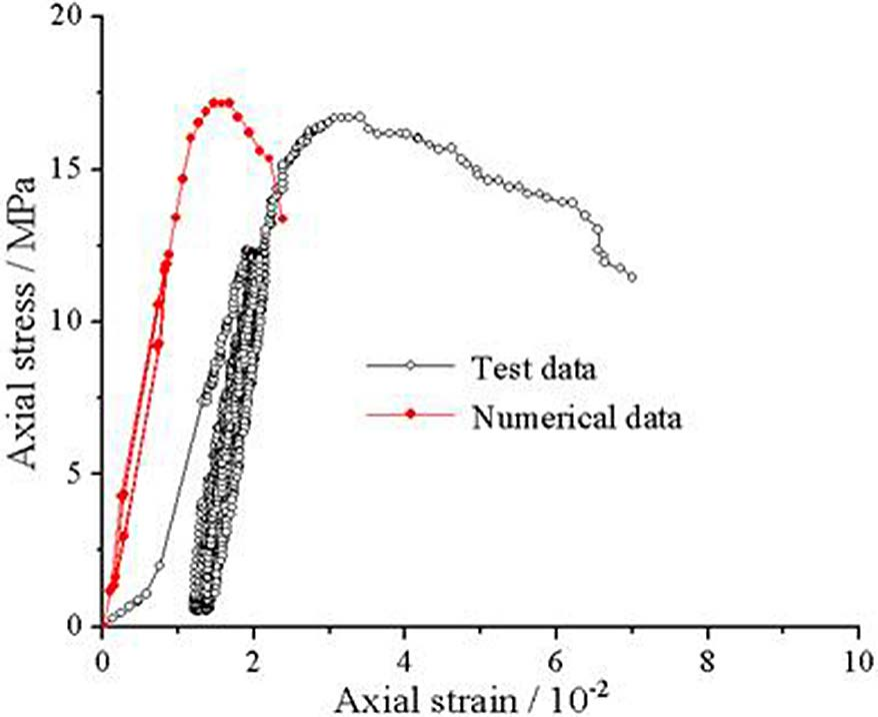
Comparison results of the axial stress–strain curves obtained from the experiment and numerical simulation under biaxial-compression cyclic loading and unloading conditions.

The mechanical properties of the specimen with a single group of cross cracks under biaxial compression cyclic loading and unloading are numerically simulated. Angle *α* of the specimen is 0°, angle *β* is 45°, and the lateral pressure is 1 MPa. Fig 17 shows the comparison results of the axial stress–strain curves obtained from the test and numerical simulation.

The stress–strain curves of the indoor test results agree well with the numerical simulation test results. Thus, the PFC numerical calculation model and calculation parameters presented in this paper can be used to perform calculations under biaxial compression cyclic loading and unloading conditions.

The numerical simulation values of the compressive strength of the samples that contain a single group of cross cracks after five and ten times of biaxial compression cyclic loading and unloading are listed in Table 9. The numerical simulation values of the compressive strength of the samples that contain multiple groups of cross cracks after five times of biaxial-compression cyclic loading and unloading are listed in Table 10.

**Table 9:** Numerical-simulation values of the compressive strength of the specimen that contains a single group of cross cracks after five times of biaxial-compression cyclic loading and unloading (MPa).

Inclined angle *α* of the main crack*/* °	Lateral stress is 1 MPa									
	after 5 times of biaxial compression cycle loading and unloading					after 10 times of biaxial compression cycle loading and unloading				
	The angle *β* between the main and the secondary cracks *β/* °					The angle *β* between the main and the secondary cracks *β/* °				
	0°	30°	45°	60°	90°	0°	30°	45°	60°	90°
0°	20.66	19.64	17.97	17.39	18.72	20.56	19.14	17.47	17.29	18.62
30°	16.82	16.38	15.25	16.47	15.50	16.52	16.28	15.15	16.37	15.41
45°	15.37	14.60	14.98	14.85	15.35	15.07	14.50	14.98	14.75	15.55
60°	14.99	12.55	14.46	12.52	13.32	14.79	12.35	14.36	12.62	13.22
90°	15.88	14.99	15.37	15.71	16.00	15.58	14.89	15.37	15.61	15.90

**Table 10:** Numerical simulation values of the compressive strength of the specimen that contains multiple groups of cross cracks after five times of biaxial compression cycle loading and unloading (MPa).

Inclined angle *α* of the main crack*/* °	Lateral stress is 1 MPa		
	Number of cross cracks		
	Two groups of cross cracks	Three groups of cross cracks	Four groups of cross cracks
0°	18.94	17.26	16.21
30°	15.72	15.14	15.45
45°	14.14	12.90	12.73
60°	12.38	12.09	11.35
90°	13.69	14.98	15.78

As per the numerical simulation results, when both *α*, the number of cross cracks, and the lateral pressure are the same, the compressive strength of the specimen after five cycles of loading and unloading is less than that of the biaxial compression. The compressive strength of the sample after 10 cycles of loading and unloading is less than that after five cycles of loading and unloading. The rule is the same as that obtained from the experiment.

## 6 Conclusions

(1) When both angle *β* between the primary and secondary prefabricated cracks and the lateral pressure are the same, the compressive strength of the specimen first decreases and then increases with increase in angle *α* from 0° to 90°. The compressive strength of the sample reaches the minimum value when *α* = 60°. When both *α* of the main prefabricated crack and the lateral pressure are the same, the compressive strength of the specimen does not significantly change with the increase in *β* from 0° to 90°. When both inclined angle *α* of the main prefabricated crack, angle *β* between the main and secondary prefabricated cracks, and the lateral pressure are the same, the compressive strength of the specimen after cyclic loading and unloading gradually decreases with increase in the number of cyclic loading and unloading.
(2) When both *α* and the lateral pressure are the same, the compressive strength of the sample gradually decreases with the increase in the number of cross cracks in the specimen. When both *α*, the number of cross cracks, and the lateral pressure are the same, the compressive strength of the specimen after cyclic loading and unloading gradually decreases with the increase in the cyclic loading and unloading times.
(3) The main crack is the primary controlling factor for the strength and crack propagation of the specimens with a single group of cross cracks. Most of the specimens primarily produce wing cracks at the end of the main prefabricated crack, which propagate and penetrate with the increase in load, thus resulting in specimen failure.
(4) Compared with that in a single group of intersecting cracks, the interaction among multiple groups of intersecting cracks changes the state of the local stress field, and the crack propagation becomes more complex.
