# Supplementary material for: Study on the mechanical properties of rock with cross fracture under biaxial compression cyclic loading and unloading
Source: PLoS One. 2026 Aug 14;21(8):e0352591. doi: 10.1371/journal.pone.0352591 (PMC13475898; doi:10.1371/journal.pone.0352591)
Supplement: Supplementary file 1 [file pone.0352591.s001.doc]

**To reviewer #1**

1. In this work, the 325 Portland cement is used as an artificial rock-like material to investigate the mechanical behavior of rock. Please explain the differences between cement and rock materials and the rationale for using this alternative in Section 2.1.

Added explanation in Section 2.1.

2. Why the crack length is set as 40 mm and 20 mm? What is the crack width? IF possible, please provide some sample images in the manuscript.

Added explanation in Section 2.1.

3. The introduction part of this paper should be significantly improved in four key aspects. First, more engineering background needs to be supplemented, as well as the potential significance and application of this study. Second, the literature review should be restructured to provide a more comprehensive and synthesized critical discussion, moving beyond a simple listing of previous work. Third, it is essential to incorporate a more up-to-date review on crack evolution and rock biaxial compression tests, particularly citing recent research frontiers from the past five years, such as 10.1016/j.enggeo.2025.108245, 10.1016/j.tust.2022.104914, 10.1016/j.ijmst.2025.11.012, and 10.1016/j.tafmec.2025.105345. Fourth, the manuscript would benefit from a clearer statement of the research gap.

We have added an engineering background explanation in the Introduction, emphasizing that the rock mass is subjected to bi-directional stress. We have also clarified why cyclic loading and unloading are crucial for tunnel engineering. However, relatively few studies published in the past five years have focused on crack evolution in rocks under biaxial compression s. The evolution law of rock cracks is related to the distribution of cracks, so it is difficult to summarize the evolution law of rocks in these literatures.

4. The quality and resolution of some figures need to be improved, such as Fig. 5.

Authors have left Chengdu University of Technology and it is inconvenient to take photos of the instrument. Therefore, Fig.5 has been deleted.

5. While the experimental results are substantial, the paper currently lacks the depth of analysis required. A more thorough interpretation would be significantly improved the manuscript's overall quality.

Added explanation

6. What is the purpose of numerical simulation? At the current stage, there are no additional information or findings obtained by the numerical model. More in-depth mechanism analysis is needed. In addition, the manuscript lacks the model parameters and model setting details.

The purpose of numerical simulation is to reproduce the mechanical properties of rocks with fractures under biaxial compression cyclic loading and unloading conditions. Since the simulation conditions were established according to the experimental conditions, no additional information or discoveries were obtained through numerical simulation. There is already information about the model parameters and model settings in section 4.

7. There are too many tables in this paper. Using some figures may be more intuitive.

There are already many images in the article. Converting many tables into images would further increase the number of images in the article. Therefore, the revised draft does not convert the table into a graph.

**To reviewer #2**

1. The literature review is adequate but could be strengthened by more explicitly linking previous studies on parallel or single-flaw specimens to the knowledge gap regarding multiple cross fractures under cyclic loading. Briefly emphasizing why cyclic loading-unloading is critical for tunnel engineering (e.g., stress redistribution after excavation, repeated traffic or blasting loads) would enhance motivation.

We have added an explanation in the Introduction to show why cyclic loading and unloading are crucial for tunnel engineering.

2. Section 2.1: Please specify the curing time and conditions for cement mortar specimens after crack insertion.

Added explanation in section 2.1

3. Section 2.2: The description of crack configurations for multiple groups (Figs. 2–4) is clear, but the choice of specific β values for different crack-group numbers (β=30° for 2 groups, 60° for 3 groups, 90° for 4 groups) should be briefly justified.

Due to the variety of experimental configurations, in order to reduce the number of test conditions, *β* containing two sets of intersecting cracks, three sets of intersecting cracks, and four sets of intersecting cracks will be temporarily randomly selected.

4. Section 2.4: Clarify whether lateral pressure was also cycled or held constant during axial cyclic loading-unloading. The text implies it was constant, but this should be stated explicitly.

The font in Fig. 1 has been reduced in size.

During cycle loading and unloading biaxial compression, the lateral pressure is maintained at 1MPa or 2MPa. It has been explained in Section 2.4. The font size in Figure 1 has been enlarged.

5. The trends in compressive strength with α and β are well documented. However, the discussion on why strength is minimized at α=60° could be elaborated slightly, possibly linking to shear stress maximization along the main crack.

Already explained in Section 3.1

6. For multiple crack groups, the statement that “crack propagation is more complex” is supported by images but would benefit from a brief mechanistic explanation—e.g., how stress field interactions alter crack initiation paths.

How stress-field interactions change the initiation path can only be determined through numerical simulation, and continuous finite element numerical simulation methods are required. Therefore, the revised manuscript does not currently provide a brief mechanical explanation, and therefore does not explain how stress field interactions alter crack initiation paths.

7. The numerical simulation validation (Figs. 16 & 17) is convincing. Consider adding a sentence on the limitations of the PFC2D model (e.g., 2D simplification, bond parameters selection) for completeness.

Added explanation in Section 4

8. The manuscript is generally well-written, but there are minor grammatical errors and occasional awkward phrasing (e.g., “fracture diagram” could be “failure pattern” or “crack propagation image”). A careful proofreading is recommended.

I have changed 'fracture diagram' to 'failure pattern'. The entire article has been revised.

9.Some figure captions (e.g., Figs. 7–11) list (a) to (e) but the labels in the images are not clearly visible in the provided text. Ensure all sub-figures are properly labeled in the final version.

The font in the labels in Figs. 7-11 is too small to display. Therefore, the revised manuscript cannot display the words in the label in the image.

10. In Tables 2–5 and 7–10, consistent use of significant figures would improve readability.

The manuscript already have many images. Converting many tables into images will increase the number of images in the article. Therefore, the revised draft does not convert the table into a graph.

11. References are relevant and up-to-date. Ensure all in-text citations are included in the reference list and vice versa

All references mentioned in the article are included in the literature list.
