# Supplementary material for: Study on the mechanical properties of rock with cross fracture under biaxial compression cyclic loading and unloading
Source: PLoS One. 2026 Aug 14;21(8):e0352591. doi: 10.1371/journal.pone.0352591 (PMC13475898; doi:10.1371/journal.pone.0352591)
Supplement: Peer review history

# Peer review: Decision Letter 0

**DOI:** 10.1371/journal.pone.0352591.r001

**Authors:** Dajiang Geng

23 Feb 2026

Dear Dr. Wang,

Thank you for submitting your manuscript to PLOS ONE. After careful consideration, we feel that it has merit but does not fully meet PLOS ONE’s publication criteria as it currently stands. Therefore, we invite you to submit a revised version of the manuscript that addresses the points raised during the review process.

Please submit your revised manuscript by Apr 09 2026 11:59PM. If you will need more time than this to complete your revisions, please reply to this message or contact the journal office at plosone@plos.org. When you're ready to submit your revision, log on to https://www.editorialmanager.com/pone/ and select the 'Submissions Needing Revision' folder to locate your manuscript file.

- A letter that responds to each point raised by the academic editor and reviewer(s). You should upload this letter as a separate file labeled 'Response to Reviewers'.
- A marked-up copy of your manuscript that highlights changes made to the original version. You should upload this as a separate file labeled 'Revised Manuscript with Track Changes'.
- An unmarked version of your revised paper without tracked changes. You should upload this as a separate file labeled 'Manuscript'.

If you would like to make changes to your financial disclosure, please include your updated statement in your cover letter. Guidelines for resubmitting your figure files are available below the reviewer comments at the end of this letter.

If applicable, we recommend that you deposit your laboratory protocols in protocols.io to enhance the reproducibility of your results. Protocols.io assigns your protocol its own identifier (DOI) so that it can be cited independently in the future. For instructions see: https://journals.plos.org/plosone/s/submission-guidelines#loc-laboratory-protocols. Additionally, PLOS ONE offers an option for publishing peer-reviewed Lab Protocol articles, which describe protocols hosted on protocols.io. Read more information on sharing protocols at https://plos.org/protocols?utm_medium=editorial-email&utm_source=authorletters&utm_campaign=protocols.

We look forward to receiving your revised manuscript.

Kind regards,

Dajiang Geng

Academic Editor

PLOS One

Journal Requirements:

When submitting your revision, we need you to address these additional requirements.

1. Please ensure that your manuscript meets PLOS ONE's style requirements, including those for file naming. The PLOS ONE style templates can be found at

https://journals.plos.org/plosone/s/file?id=wjVg/PLOSOne_formatting_sample_main_body.pdf and

https://journals.plos.org/plosone/s/file?id=ba62/PLOSOne_formatting_sample_title_authors_affiliations.pdf

2. Please note that PLOS One has specific guidelines on code sharing for submissions in which author-generated code underpins the findings in the manuscript. In these cases, we expect all author-generated code to be made available without restrictions upon publication of the work. Please review our guidelines at https://journals.plos.org/plosone/s/materials-and-software-sharing#loc-sharing-code and ensure that your code is shared in a way that follows best practice and facilitates reproducibility and reuse.

3. Thank you for stating the following in the Acknowledgments Section of your manuscript:

“This work was supported by the Open Research Fund of State Key Laboratory of Geohazard Prevention and Geoenvironment Protection (Grant no. SKLGP2021K020) and the Open Research Fund of Engineering Research Center of Underground Mine Construction, Ministry of Education (Grant No. JYBGCZX2020101)”

We note that you have provided funding information that is not currently declared in your Funding Statement. However, funding information should not appear in the Acknowledgments section or other areas of your manuscript. We will only publish funding information present in the Funding Statement section of the online submission form.

Please remove any funding-related text from the manuscript and let us know how you would like to update your Funding Statement. Currently, your Funding Statement reads as follows:

“This work was supported by the Open Research Fund of State Key Laboratory of Geohazard Prevention and Geoenvironment Protection (Grant no. SKLGP2021K020) and the Open Research Fund of Engineering Research Center of Underground Mine Construction, Ministry of Education (Grant No. JYBGCZX2020101).”

Please include your amended statements within your cover letter; we will change the online submission form on your behalf.

4. Thank you for stating the following financial disclosure:

“This work was supported by the Open Research Fund of State Key Laboratory of Geohazard Prevention and Geoenvironment Protection (Grant no. SKLGP2021K020) and the Open Research Fund of Engineering Research Center of Underground Mine Construction, Ministry of Education (Grant No. JYBGCZX2020101).”

Please state what role the funders took in the study.  If the funders had no role, please state: "The funders had no role in study design, data collection and analysis, decision to publish, or preparation of the manuscript."

If this statement is not correct you must amend it as needed.

Please include this amended Role of Funder statement in your cover letter; we will change the online submission form on your behalf.

5. Please provide a complete Data Availability Statement in the submission form, ensuring you include all necessary access information or a reason for why you are unable to make your data freely accessible. If your research concerns only data provided within your submission, please write "All data are in the manuscript and/or supporting information files" as your Data Availability Statement.

6. PLOS requires an ORCID iD for the corresponding author in Editorial Manager on papers submitted after December 6th, 2016. Please ensure that you have an ORCID iD and that it is validated in Editorial Manager. To do this, go to ‘Update my Information’ (in the upper left-hand corner of the main menu), and click on the Fetch/Validate link next to the ORCID field. This will take you to the ORCID site and allow you to create a new iD or authenticate a pre-existing iD in Editorial Manager.

7. If the reviewer comments include a recommendation to cite specific previously published works, please review and evaluate these publications to determine whether they are relevant and should be cited. There is no requirement to cite these works unless the editor has indicated otherwise.

[Note: HTML markup is below. Please do not edit.]

Reviewers' comments:

Reviewer's Responses to Questions

**Comments to the Author**

1. Is the manuscript technically sound, and do the data support the conclusions?

Reviewer #1: Yes

Reviewer #2: Yes

**********

2. Has the statistical analysis been performed appropriately and rigorously?

Reviewer #1: Yes

Reviewer #2: Yes

**********

3. Have the authors made all data underlying the findings in their manuscript fully available?

The PLOS Data policy

Reviewer #1: Yes

Reviewer #2: Yes

**********

4. Is the manuscript presented in an intelligible fashion and written in standard English?

Reviewer #1: Yes

Reviewer #2: Yes

**********

Reviewer #1: This manuscript investigates the mechanical properties of rock with cross fracture via biaxial compression cyclic loading and unloading tests on rock-like samples. The study yielded a robust body of experimental results. At present, the quality of the manuscript does not meet the publishing standard, and a major revision is necessary major revision is required.

1. In this work, the 325 Portland cement is used as an artificial rock-like material to investigate the mechanical behavior of rock. Please explain the differences between cement and rock materials and the rationale for using this alternative in Section 2.1.

2. Why the crack length is set as 40 mm and 20 mm? What is the crack width? IF possible, please provide some sample images in the manuscript.

3. The introduction part of this paper should be significantly improved in four key aspects. First, more engineering background needs to be supplemented, as well as the potential significance and application of this study. Second, the literature review should be restructured to provide a more comprehensive and synthesized critical discussion, moving beyond a simple listing of previous work. Third, it is essential to incorporate a more up-to-date review on crack evolution and rock biaxial compression tests, particularly citing recent research frontiers from the past five years, such as 10.1016/j.enggeo.2025.108245, 10.1016/j.tust.2022.104914, 10.1016/j.ijmst.2025.11.012, and 10.1016/j.tafmec.2025.105345. Fourth, the manuscript would benefit from a clearer statement of the research gap.

4. The quality and resolution of some figures need to be improved, such as Fig. 5.

5. While the experimental results are substantial, the paper currently lacks the depth of analysis required. A more thorough interpretation would be significantly improved the manuscript's overall quality.

6. What is the purpose of numerical simulation? At the current stage, there are no additional information or findings obtained by the numerical model. More in-depth mechanism analysis is needed. In addition, the manuscript lacks the model parameters and model setting details.

7. There are too many tables in this paper. Using some figures may be more intuitive.

Reviewer #2: This manuscript presents a well-structured and technically sound experimental and numerical investigation into the mechanical behavior of rock-like specimens containing single and multiple sets of cross fractures under biaxial compression and cyclic loading-unloading conditions. The study addresses a relevant engineering problem—tunnel surrounding rock stability—and systematically examines the influences of crack geometry (angles α and β), lateral pressure, number of crack groups, and cyclic loading history on compressive strength and crack evolution. The combination of physical tests and PFC2D numerical simulation adds robustness to the findings. The results are clearly presented, and the conclusions are largely supported by the data. The work is suitable for publication following minor revisions aimed at improving clarity, contextualization, and methodological detail.

Specific Comments:

(1)The literature review is adequate but could be strengthened by more explicitly linking previous studies on parallel or single-flaw specimens to the knowledge gap regarding multiple cross fractures under cyclic loading. Briefly emphasizing why cyclic loading-unloading is critical for tunnel engineering (e.g., stress redistribution after excavation, repeated traffic or blasting loads) would enhance motivation.

(2)Section 2.1: Please specify the curing time and conditions for cement mortar specimens after crack insertion.

(3)Section 2.2: The description of crack configurations for multiple groups (Figs. 2–4) is clear, but the choice of specific β values for different crack-group numbers (β=30° for 2 groups, 60° for 3 groups, 90° for 4 groups) should be briefly justified.

(4)Section 2.4: Clarify whether lateral pressure was also cycled or held constant during axial cyclic loading-unloading. The text implies it was constant, but this should be stated explicitly.

�5�The trends in compressive strength with α and β are well documented. However, the discussion on why strength is minimized at α=60° could be elaborated slightly, possibly linking to shear stress maximization along the main crack.

�6�For multiple crack groups, the statement that “crack propagation is more complex” is supported by images but would benefit from a brief mechanistic explanation—e.g., how stress field interactions alter crack initiation paths.

(7) The numerical simulation validation (Figs. 16 & 17) is convincing. Consider adding a sentence on the limitations of the PFC2D model (e.g., 2D simplification, bond parameters selection) for completeness.

(8)The manuscript is generally well-written, but there are minor grammatical errors and occasional awkward phrasing (e.g., “fracture diagram” could be “failure pattern” or “crack propagation image”). A careful proofreading is recommended.

(9)Some figure captions (e.g., Figs. 7–11) list (a) to (e) but the labels in the images are not clearly visible in the provided text. Ensure all sub-figures are properly labeled in the final version.

(10)In Tables 2–5 and 7–10, consistent use of significant figures would improve readability.

(11)References are relevant and up-to-date. Ensure all in-text citations are included in the reference list and vice versa

**********

what does this mean?). If published, this will include your full peer review and any attached files.

If you choose “no”, your identity will remain anonymous but your review may still be made public.

**Do you want your identity to be public for this peer review?**  For information about this choice, including consent withdrawal, please see our Privacy Policy

Reviewer #1: No

Reviewer #2: No

**********

[NOTE: If reviewer comments were submitted as an attachment file, they will be attached to this email and accessible via the submission site. Please log into your account, locate the manuscript record, and check for the action link "View Attachments". If this link does not appear, there are no attachment files.]

To ensure your figures meet our technical requirements, please review our figure guidelines: https://journals.plos.org/plosone/s/figures

You may also use PLOS’s free figure tool, NAAS, to help you prepare publication quality figures: https://journals.plos.org/plosone/s/figures#loc-tools-for-figure-preparation.

NAAS will assess whether your figures meet our technical requirements by comparing each figure against our figure specifications.

# Author comment: Author response to Decision Letter 1

**DOI:** 10.1371/journal.pone.0352591.r002

13 May 2026

To reviewer #1

1. In this work, the 325 Portland cement is used as an artificial rock-like material to investigate the mechanical behavior of rock. Please explain the differences between cement and rock materials and the rationale for using this alternative in Section 2.1.

Added explanation in Section 2.1.

2. Why the crack length is set as 40 mm and 20 mm? What is the crack width? IF possible, please provide some sample images in the manuscript.

Added explanation in Section 2.1.

3. The introduction part of this paper should be significantly improved in four key aspects. First, more engineering background needs to be supplemented, as well as the potential significance and application of this study. Second, the literature review should be restructured to provide a more comprehensive and synthesized critical discussion, moving beyond a simple listing of previous work. Third, it is essential to incorporate a more up-to-date review on crack evolution and rock biaxial compression tests, particularly citing recent research frontiers from the past five years, such as 10.1016/j.enggeo.2025.108245, 10.1016/j.tust.2022.104914, 10.1016/j.ijmst.2025.11.012, and 10.1016/j.tafmec.2025.105345. Fourth, the manuscript would benefit from a clearer statement of the research gap.

We have added an engineering background explanation in the Introduction, emphasizing that the rock mass is subjected to bi-directional stress. We have also clarified why cyclic loading and unloading are crucial for tunnel engineering. However, relatively few studies published in the past five years have focused on crack evolution in rocks under biaxial compression s. The evolution law of rock cracks is related to the distribution of cracks, so it is difficult to summarize the evolution law of rocks in these literatures.

4. The quality and resolution of some figures need to be improved, such as Fig. 5.

Authors have left Chengdu University of Technology and it is inconvenient to take photos of the instrument. Therefore, Fig.5 has been deleted.

5. While the experimental results are substantial, the paper currently lacks the depth of analysis required. A more thorough interpretation would be significantly improved the manuscript's overall quality.

Added explanation

6. What is the purpose of numerical simulation? At the current stage, there are no additional information or findings obtained by the numerical model. More in-depth mechanism analysis is needed. In addition, the manuscript lacks the model parameters and model setting details.

The purpose of numerical simulation is to reproduce the mechanical properties of rocks with fractures under biaxial compression cyclic loading and unloading conditions. Since the simulation conditions were established according to the experimental conditions, no additional information or discoveries were obtained through numerical simulation. There is already information about the model parameters and model settings in section 4.

7. There are too many tables in this paper. Using some figures may be more intuitive.

There are already many images in the article. Converting many tables into images would further increase the number of images in the article. Therefore, the revised draft does not convert the table into a graph.

To reviewer #2

1. The literature review is adequate but could be strengthened by more explicitly linking previous studies on parallel or single-flaw specimens to the knowledge gap regarding multiple cross fractures under cyclic loading. Briefly emphasizing why cyclic loading-unloading is critical for tunnel engineering (e.g., stress redistribution after excavation, repeated traffic or blasting loads) would enhance motivation.

We have added an explanation in the Introduction to show why cyclic loading and unloading are crucial for tunnel engineering.

2. Section 2.1: Please specify the curing time and conditions for cement mortar specimens after crack insertion.

Added explanation in section 2.1

3. Section 2.2: The description of crack configurations for multiple groups (Figs. 2–4) is clear, but the choice of specific β values for different crack-group numbers (β=30° for 2 groups, 60° for 3 groups, 90° for 4 groups) should be briefly justified.

Due to the variety of experimental configurations, in order to reduce the number of test conditions, β containing two sets of intersecting cracks, three sets of intersecting cracks, and four sets of intersecting cracks will be temporarily randomly selected.

4. Section 2.4: Clarify whether lateral pressure was also cycled or held constant during axial cyclic loading-unloading. The text implies it was constant, but this should be stated explicitly.

The font in Fig. 1 has been reduced in size.

During cycle loading and unloading biaxial compression, the lateral pressure is maintained at 1MPa or 2MPa. It has been explained in Section 2.4. The font size in Figure 1 has been enlarged.

5. The trends in compressive strength with α and β are well documented. However, the discussion on why strength is minimized at α=60° could be elaborated slightly, possibly linking to shear stress maximization along the main crack.

Already explained in Section 3.1

6. For multiple crack groups, the statement that “crack propagation is more complex” is supported by images but would benefit from a brief mechanistic explanation—e.g., how stress field interactions alter crack initiation paths.

How stress-field interactions change the initiation path can only be determined through numerical simulation, and continuous finite element numerical simulation methods are required. Therefore, the revised manuscript does not currently provide a brief mechanical explanation, and therefore does not explain how stress field interactions alter crack initiation paths.

7. The numerical simulation validation (Figs. 16 & 17) is convincing. Consider adding a sentence on the limitations of the PFC2D model (e.g., 2D simplification, bond parameters selection) for completeness.

Added explanation in Section 4

8. The manuscript is generally well-written, but there are minor grammatical errors and occasional awkward phrasing (e.g., “fracture diagram” could be “failure pattern” or “crack propagation image”). A careful proofreading is recommended.

I have changed 'fracture diagram' to 'failure pattern'. The entire article has been revised.

9.Some figure captions (e.g., Figs. 7–11) list (a) to (e) but the labels in the images are not clearly visible in the provided text. Ensure all sub-figures are properly labeled in the final version.

The font in the labels in Figs. 7-11 is too small to display. Therefore, the revised manuscript cannot display the words in the label in the image.

10. In Tables 2–5 and 7–10, consistent use of significant figures would improve readability.

The manuscript already have many images. Converting many tables into images will increase the number of images in the article. Therefore, the revised draft does not convert the table into a graph.

11. References are relevant and up-to-date. Ensure all in-text citations are included in the reference list and vice versa

All references mentioned in the article are included in the literature list.

Attachment Submitted filename: Response to Reviewers.doc

# Peer review: Decision Letter 1

**DOI:** 10.1371/journal.pone.0352591.r003

**Authors:** Dajiang Geng

11 Jun 2026

Study on the mechanical properties of rock with cross fracture under biaxial compression cyclic loading and unloading

PONE-D-26-03904R1

Dear Dr. Hanqiang Wang,

We’re pleased to inform you that your manuscript has been judged scientifically suitable for publication and will be formally accepted for publication once it meets all outstanding technical requirements.

Within one week, you’ll receive an e-mail detailing the required amendments. When these have been addressed, you’ll receive a formal acceptance letter and your manuscript will be scheduled for publication.

An invoice will be generated when your article is formally accepted. Please note, if your institution has a publishing partnership with PLOS and your article meets the relevant criteria, all or part of your publication costs will be covered. Please make sure your user information is up-to-date by logging into Editorial Manager at Editorial Manager® and clicking the ‘Update My Information' link at the top of the page. For questions related to billing, please contact billing support.

If your institution or institutions have a press office, please notify them about your upcoming paper to help maximize its impact. If they’ll be preparing press materials, please inform our press team as soon as possible -- no later than 48 hours after receiving the formal acceptance. Your manuscript will remain under strict press embargo until 2 pm Eastern Time on the date of publication. For more information, please contact onepress@plos.org.

Kind regards,

Dajiang Geng

Academic Editor

PLOS One

Additional Editor Comments (optional):

Reviewers' comments:

Reviewer's Responses to Questions

**Comments to the Author**

Reviewer #3: All comments have been addressed

**********

2. Is the manuscript technically sound, and do the data support the conclusions?

Reviewer #3: Yes

**********

3. Has the statistical analysis been performed appropriately and rigorously?

Reviewer #3: Yes

**********

4. Have the authors made all data underlying the findings in their manuscript fully available?

The PLOS Data policy

Reviewer #3: Yes

**********

5. Is the manuscript presented in an intelligible fashion and written in standard English?

Reviewer #3: Yes

**********

Reviewer #3: The author has fully addressed all comments raised in the review. The manuscript is now acceptable for publication.

**********

what does this mean?). If published, this will include your full peer review and any attached files.

If you choose “no”, your identity will remain anonymous but your review may still be made public.

**Do you want your identity to be public for this peer review?**  For information about this choice, including consent withdrawal, please see our Privacy Policy

Reviewer #3: No

**********

# Editor report: Acceptance letter

**DOI:** 10.1371/journal.pone.0352591.r004

**Authors:** Dajiang Geng

PONE-D-26-03904R1

PLOS One

Dear Dr. Wang,

I'm pleased to inform you that your manuscript has been deemed suitable for publication in PLOS One. Congratulations! Your manuscript is now being handed over to our production team.

At this stage, our production department will prepare your paper for publication. This includes ensuring the following:

* All references, tables, and figures are properly cited

* All relevant supporting information is included in the manuscript submission,

* There are no issues that prevent the paper from being properly typeset

You will receive further instructions from the production team, including instructions on how to review your proof when it is ready. Please keep in mind that we are working through a large volume of accepted articles, so please give us a few days to review your paper and let you know the next and final steps.

Lastly, if your institution or institutions have a press office, please let them know about your upcoming paper now to help maximize its impact. If they'll be preparing press materials, please inform our press team within the next 48 hours. Your manuscript will remain under strict press embargo until 2 pm Eastern Time on the date of publication. For more information, please contact onepress@plos.org.

You will receive an invoice from PLOS for your publication fee after your manuscript has reached the completed accept phase. If you receive an email requesting payment before acceptance or for any other service, this may be a phishing scheme. Learn how to identify phishing emails and protect your accounts at https://explore.plos.org/phishing.

If we can help with anything else, please email us at customercare@plos.org.

Thank you for submitting your work to PLOS ONE and supporting open access.

Kind regards,

PLOS ONE Editorial Office Staff

on behalf of

Dr. Dajiang Geng

Academic Editor

PLOS One
